# FOXO1 inhibits the invasion and metastasis of hepatocellular carcinoma by reversing ZEB2-induced epithelial-mesenchymal transition

**DOI:** 10.18632/oncotarget.13786

**Published:** 2016-12-03

**Authors:** Tianxiu Dong, Yu Zhang, Yaodong Chen, Pengfei Liu, Tingting An, Jiuwei Zhang, Haichao Yang, Wenjing Zhu, Xiuhua Yang

**Affiliations:** ^1^ Department of Abdominal Ultrasound, The First Affiliated Hospital of Harbin Medical University, Harbin 150001, China; ^2^ Department of Magnetic Resonance Imaging, The First Affiliated Hospital of Harbin Medical University, Harbin 150001, China

**Keywords:** FOXO1, hepatocellular carcinoma, epithelial-mesenchymal transition, ZEB2, TGF-β

## Abstract

The epithelial-to-mesenchymal transition (EMT) program is critical for epithelial cell cancer progression and fibrotic diseases. FOXO1 influences a broad range of physiological and pathological processes. However, the mechanism by which FOXO1 inhibits EMT is not fully understood. In this study, we demonstrated that FOXO1 overexpression inhibited cell motility and invasiveness *in vitro* and inhibited lung metastasis *in vivo.* In addition, we found that FOXO1 couldreverse the EMT program. FOXO1 silencing by siRNA in hepatocellular carcinoma (HCC) cell lines enhanced the expression of mesenchymal markers and decreased the expression of the epithelial markers. Consistent with these findings, FOXO1 overexpression exerted opposite effects. Furthermore, we found that FOXO1 levels were inversely correlated with the levels of EMT inducers, including Snail, Slug, ZEB1, ZEB2 and Twist1 in HCC cells. Co-immunoprecipitation and immunohistochemistry assays revealed that an interaction between FOXO1 and ZEB2. A dual-luciferase reporter assay and a ChIP assay further demonstrated that FOXO1 binds to the ZEB2 promoter. Together, these findings suggest that FOXO1 overexpression or ZEB2 inhibition might be potential therapeutic strategies for treating HCC.

## INTRODUCTION

Hepatocellular carcinoma (HCC) is one of the most common solid malignancies worldwide and is the second leading cause of cancer-related mortality in Asia [1]. The prognosis of HCC remains poor due to the lack of effective treatment options and the high probability of metastasis and recurrence after surgical resection [2]. In addition, the molecular mechanisms underlying HCC metastasis remain poorly characterized. Thus, identifying the mechanisms that mediate HCC metastasis is imperative to improving outcomes.

Epithelial-mesenchymal transition (EMT) plays a vital role in the progression of metastasis in multiple cancers by inducing epithelial cells to adopt mesenchymal attributes [3]. During this process, well-organized and tightly connected epithelial cells differentiate into disorganized and motile mesenchymal cells [4]. The EMT is regulated by a complex network of interconnected pathways controlled by TGF-β signaling [5]. EMT-inducing transcription factors (EMT-TFs) include the Snail family protein members Snail1 and Snail2 (also referred to as Slug), the two-handed zinc finger factors ZEB1 and ZEB2, and the basic helix-loop-helix (bHLH) protein Twist1 [6]. ZEB2 plays a key role in the TGF-β signaling cascade and promotes tumor cell invasion and metastasis [7]. Snail, Slug, ZEB1, ZEB2 and Twist1 are all potent repressors of E-cadherin [8–11]. Determining the structure and functional dynamics of these factors is critical to further characterizing the pathogenesis of EMT in HCC progression.

The FoxO family of transcription factors (FoxOs), FOXO1 (FKHR), FOXO3 (FKHRL1 or FOXO3a), FOXO4 (AFX) and FOXO6, integrate multiple cellular signals and translate various environmental stimuli into dynamic patterns of gene expression that influence a broad range of physiological and pathological processes, including cancer and aging [12]. FoxOs are associated with the insulin/IGF and growth factor-activated receptor tyrosine kinase (RTK)-phosphoinostide-3-kinase (PI3K) signaling pathway [13]. FOXO1 dysregulation has been observed in several human cancers, and this aberration influences multiple cellular functions, including apoptosis, cell cycle control, DNA damage repair, carcinogenesis, glucose metabolism and tumor immunity [14–16]. Furthermore, cancer stem cells (CSCs) in pancreatic ductal carcinoma are FOXO1-negative, and the loss of FOXO1 might be a more accurate CSC marker than the expression of CD133 or ALDH1 [17]. A recent report demonstrated that FOXO1 regulates the vascular growth that couples metabolic and proliferative activities in endothelial cells [18]. However, the impact of FOXO1 on invasion and metastasis in HCC is poorly understood. In addition, the underlying mechanisms that promote HCC invasion and metastasis remain elusive. Thus, we sought to investigate the mechanisms by which FOXO1 inhibits HCC migration and invasion.

In this study, we provide the first evidence that FOXO1 can reverse EMT in HCC via the transcription inducers Snail, Slug, ZEB1, ZEB2 and Twist1, with ZEB2 playing a particularly critical role in this process. Furthermore, we demonstrated that FOXO1 disrupts TGF-β-induced EMT.

## RESULTS

### FOXO1 inhibits HCC cells migration and invasion

**Figure 1:**
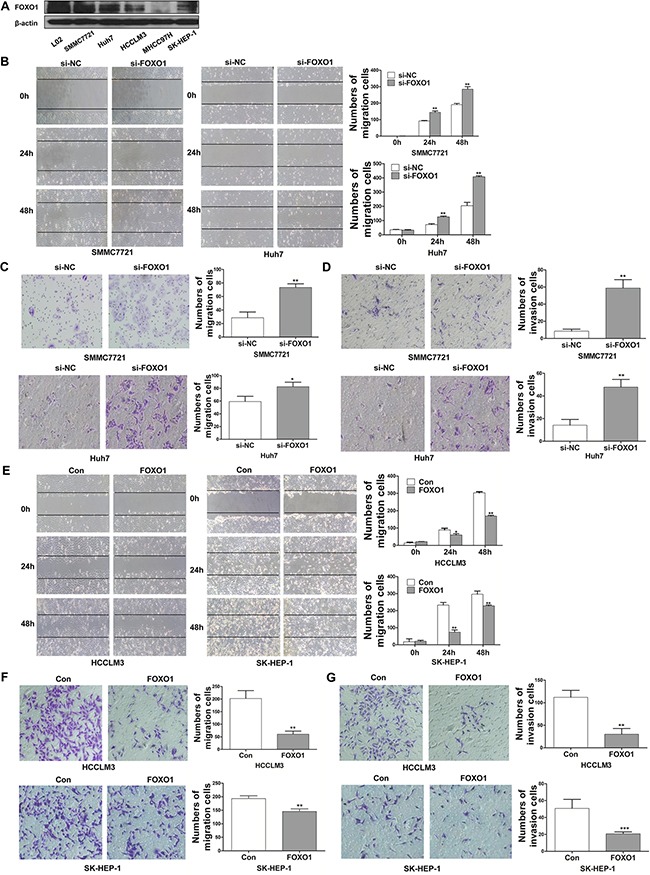
FOXO1 inhibits the migration and invasion of HCC cells **A.** Western blotting analysis of FOXO1 expression in different HCC cell lines and the normal hepatic cell line L02. **B.** Wound-healing **C.** Transwell migration assays. **D.** Matrigel invasion assays in FOXO1-silenced SMMC-7721 and Huh7 cells. **E** and **F.** Cell migration and **G.** Invasion assays in HCCLM3 and SK-HEP-1 cells overexpressing FOXO1. Cells were counted in 3 randomized fields at a magnification of 100×. The error bar represents the mean±SD of triplicate assays (*p<0.05, ** p<0.01, *** p<0.001; p-values were calculated using Student's t-test).

To explore the effects of FOXO1 on the invasiveness of HCC cells, we examined the expression of FOXO1 in five HCC cell lines (SMMC-7721, Huh7, HCCLM3, MHCC97H and SK-HEP-1), which exhibit different invasive behaviors. The expression of FOXO1 in these HCC cell lines was compared with that in a normal hepatic cell line, L02. FOXO1 protein levels were highest in normal hepatic L02 cells and were decreased in SMMC7721 and Huh7 cells (low metastatic potential), and finally, were lowest in HCCLM3, MHCC97H and SK-HEP-1 cells (high metastatic potential) (Figure 1A). To further evaluate the role of FOXO1 in HCC cells migration and invasion, we established stable cell lines that were infected with the LV-NC lentivirus (referred to as HCCLM3-Control and SK-HEP-1-Control) or with the LV-FOXO1 lentivirus (referred to as HCCLM3-FOXO1 and SK-HEP-1-FOXO1). In addition, the SMMC7721 and Huh7 cell lines were transfected with siRNA-NC or siRNA-FOXO1. A western blot analysis indicated that FOXO1 expression was up-regulated by FOXO1 overexpression and silenced by siRNA-FOXO1. FOXO1 silencing increased the wound-healing capability of SMMC7721 and Huh7 cells, while FOXO1 overexpression reduced the wound-healing capability of HCCLM3 and SK-HEP-1 cells (Figure 1B and 1E). Consistent with these findings, a Transwell assay demonstrated that FOXO1 silencing significantly enhanced the migration of SMMC7721 and Huh7 cells by 2.57-and 1.39-fold, respectively, and enhanced the invasion of these cells by 6.91- and 3.36-fold, respectively (Figure 1C and 1D). Conversely, FOXO1 overexpression markedly inhibited the migration of HCCLM3 and SK-HEP-1cells by 3.34-and 1.33-fold, respectively, and inhibited the invasion of these cells by 3.73-and 2.46-fold, respectively (Figure 1F and 1G). Together, these data indicate that FOXO1 inhibits the motility and invasiveness of HCC cells.

### FOXO1 represses epithelial-to-mesenchymal transition in HCC cells

**Figure 2:**
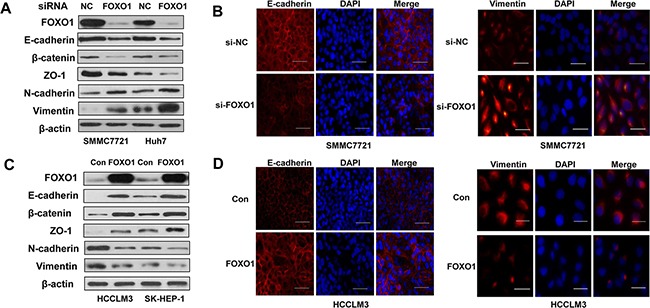
FOXO1 reverses EMT in HCC cells **A.** Western blot and **B.** Immunofluorescence staining assays revealed a down-regulation of the expression of epithelial markers (E-cadherin, β-catenin and ZO-1) and an up-regulation of the expression of mesenchymal markers (Vimentin and N-cadherin) in SMMC7721 and Huh7 cells transfected with FOXO1 siRNA. In contrast, **C.** Western blotting and **D.** Immunofluorescence staining revealed that FOXO1 overexpression up-regulated the expression of epithelial markers and decreased the expression of mesenchymal markers in HCCLM3 and SK-HEP-1 cells. Scale bar indicates 25μm.

EMT is a developmental program that converts adherent epithelial cells into mesenchymal migratory cells capable of promoting tumor metastasis. To investigate whether FOXO1 suppresses HCC invasion and migration by reversing EMT, we evaluated epithelial and mesenchymal markers in HCC cells using western blot and immunofluorescence assays. A western blot analysis confirmed that FOXO1 protein levels were down-regulated in both SMMC-7721 and Huh7 cells that were transfected with FOXO1 siRNA and were up-regulated in HCCLM3 and SK-HEP-1 cells that were infected with the LV-FOXO1 lentivirus. E-cadherin, β-catenin and ZO-1 were down-regulated in FOXO1-silenced HCC cells compared with the negative control, whereas the mesenchymal markers Vimentin and N-cadherin were strongly up-regulated (Figure 2A and 2B). FOXO1 overexpression exerted the opposite effects (Figure 2C and 2D).

### FOXO1 inhibits TGF-β-induced epithelial-to-mesenchymal transition

**Figure 3:**
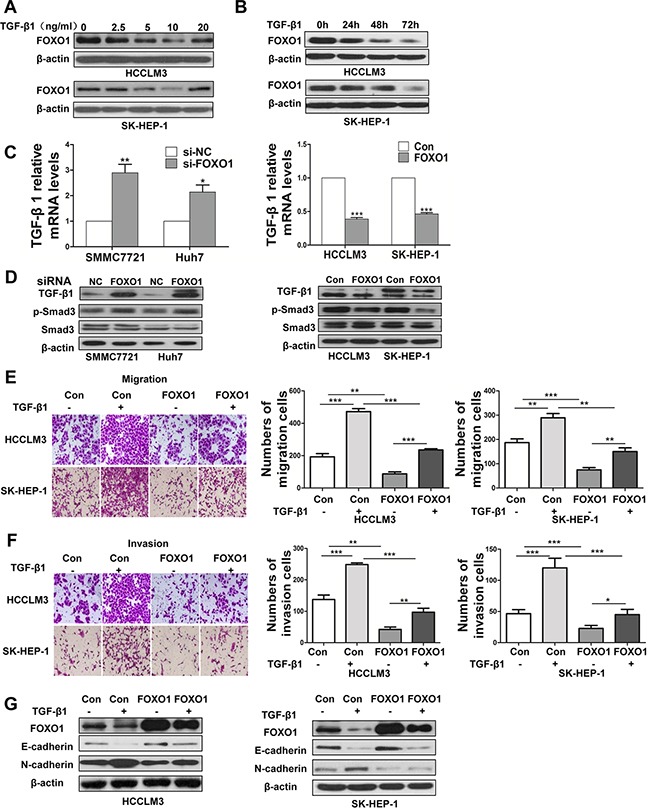
FOXO1 reverses TGF-β-induced EMT **A.** FOXO1 expression levels in HCCLM3 and SK-HEP-1 cells treated with different doses of TGF-β1. **B.** FOXO1 expression levels in HCCLM3 and SK-HEP-1 cells treated with activated TGF-β1 (10 ng/ml) at 3 time points were evaluated by western blotting. **C.** TGF-β1 mRNA levels were determined using real-time PCR in HCC cells treated with si-NC, siRNA-FOXO1, empty vector or LV-FOXO1. The error bar represents the mean±SD of triplicate assays. (*p<0.05, **p<0.01, ***p<0.001; p-values were calculated using Student's t-test). **D.** TGF-β1, p-Smad3, and Smad3 expression in HCC cell lines was analyzed by western blotting. Down-regulation of FOXO1 using siRNA markedly increased the expression of TGF-β1 and p-Smad3 in SMMC7721 and Huh7 cells. In contrast, the overexpression of FOXO1 significantly attenuated the expression of TGF-β1 and inhibited Smad3 phosphorylation. Relative changes of the number of HCCLM3 and SK-HEP-1 cells that migrated **E.** and invaded **F.** after TGF-β1 treatment or FOXO1 overexpression. (*p<0.05, **p<0.01, ***p<0.001; p-values were calculated using Student's t-test). **G.** Western blot analysis of FOXO1, E-cadherin and N-cadherin expression after treatment with TGF-β1 (10 ng/ml) in HCCLM3 and SK-HEP-1 cells that were infected with the LV-NC lentivirus or LV-FOXO1 lentivirus.

TGF-β signaling pathways are associated with the development of liver cancer [19]. We next investigated whether TGF-β signaling-induced EMT was repressed by FOXO1. A western blot analysis was used to determine the level of FOXO1 protein in response to different doses of TGF-β1 in HCCLM3 and SK-HEP-1 cells. The analysis showed that, FOXO1 expression decreased in response to low doses of TGF-β1 and that FOXO1 expression was lowest at a concentration of 10 ng/ml TGF-β1 (Figure 3A). We next evaluated the FOXO1 levels in HCCLM3 and SK-HEP-1 cells treated with 10 ng/ml TGF-β1 for 24 h, 48 h or 72 h, and observed the greatest decrease at 72 h (Figure 3B). Real-time PCR and western blotting revealed that TGF-β1 expression was increased in FOXO1-silenced HCC cell lines. In contrast, TGF-β1 expression was decreased in HCC cell lines overexpressing FOXO1 (Figure 3C and 3D). Moreover, FOXO1 silencing enhanced Smad3 phosphorylation, while FOXO1 overexpression inhibited Smad3 activation (Figure 3D). These data indicate that TGF-β1 and FOXO1 each repress the expression of the other. HCC cells treated with TGF-β1 led to a promotion of cell migration and invasion, but this promotion was greatly attenuated by FOXO1 overexpression (Figure 3E and 3F). FOXO1 overexpression inhibited N-cadherin expression, and this inhibition persisted even after TGF-β1 treatment. In addition, E-cadherin expression was enhanced in the FOXO1-overexpressing cells. The FOXO1-induced increase in E-cadherin expression was inhibited by treatment with TGF-β1 (Figure 3G). These results suggest that the effects of FOXO1 associated with TGF-β1 more potently affect N-cadherin expression compared with E-cadherin expression and indicate that FOXO1 expression in HCC cells inhibits TGF-β signaling.

### FOXO1 overexpression in luciferase-labeled HCCLM3 and SK-HEP-1 cells inhibits lung metastasis in nude mice

**Figure 4:**
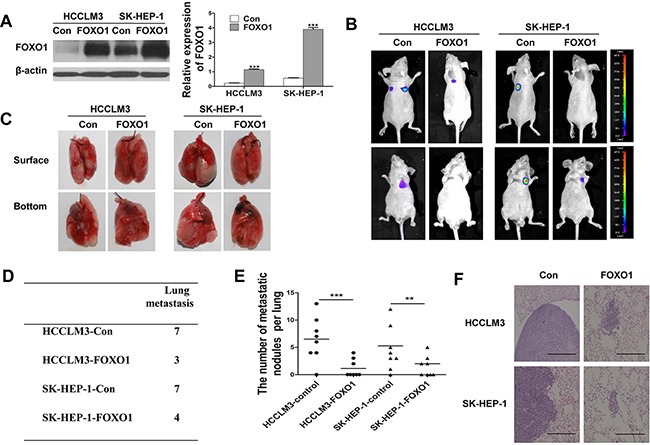
FOXO1 overexpression in luciferase-labeled HCCLM3 and SK-HEP-1 cells inhibits lung metastasis **A.** Western blot analysis of FOXO1 expression in HCCLM3 and SK-HEP-1 cells that were infected with empty vector or LV-FOXO1. (***p<0.001; p-values were calculated using Student's t-test) **B.** The 8 nude mice in each group were bioluminescently imaged 6 weeks after the injection of HCC cells, and representative images for each group are shown. **C.** Representative images of the lungs of the study mice from the *in vivo* metastasis assay. **D.** The incidence of lung metastasis in each group. **E.** The number of lung metastatic foci in each group. (**p<0.01, ***p<0.001; p-values were calculated using Student's t-test). **F.** Representative images of H&E-stained lung tissue samples from each group. Scale bar indicates 200μm.

To investigate the role of FOXO1 *in vivo*, we injected stably transfected cell lines (HCCLM3-Control, SK-HEP-1-Control, HCCLM3-FOXO1 and SK-HEP-1-FOXO1) into the tail vein of nude mice and examined the mice for the presence of lung metastatic nodules. The expression of FOXO1 was verified by western blotting, which showed that FOXO1 expression in HCCLM3-FOXO1 and SK-HEP-1-FOXO1 cells was increased approximately 5.25- and 6.93-fold, respectively, compared with the control (Figure 4A). Bioluminescent imaging of nude mice demonstrated that the incidence of lung metastasis was lower in mice injected with HCCLM3-FOXO1 or SK-HEP-1-FOXO1 cells compared with mice injected with HCCLM3-Control or SK-HEP-1-Control cells (Figure 4B). Moreover, mice injected with HCCLM3-FOXO1 or SK-HEP-1-FOXO1 cells exhibited fewer and smaller lung metastases compared with the control group (Figure 4C-4E). Histologic analyses of the dissected lungs further confirmed the presence of metastases (Figure 4F). These results demonstrate that FOXO1 plays a critical role in inhibiting HCC invasion and metastasis.

### FOXO1 negatively regulates EMT-inducing transcription factors and interacts with ZEB2 in HCC cells

**Figure 5:**
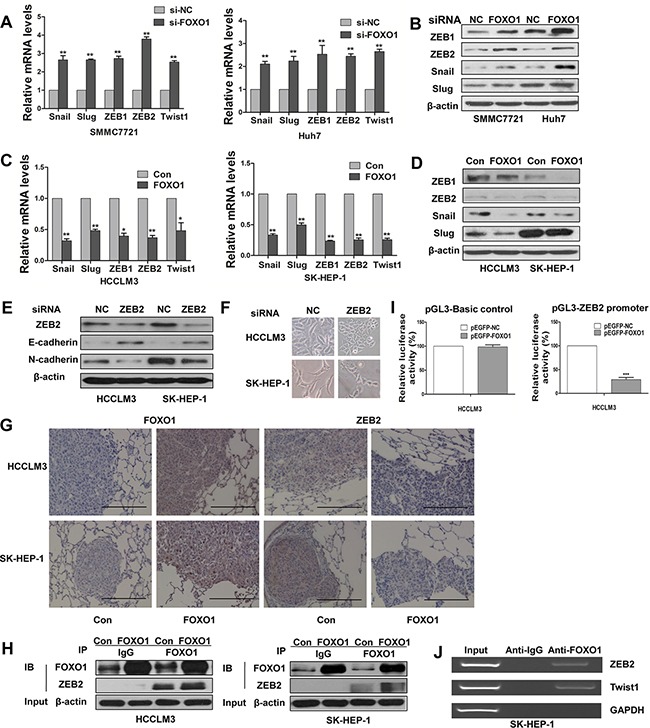
Functional effects of FOXO1 on EMT-inducing transcription factors **A.** Real-time PCR and **B.** Western blot assays demonstrate the increased expression of Snail, Slug, ZEB1, ZEB2 and Twist1 protein in SMMC7721 and Huh7 transfected with siRNA-FOXO1. **C.** Real-time PCR and **D.** Western blot assays demonstrate the expression of Snail, Slug, ZEB1, ZEB2 and Twist1 in HCCLM3 and SK-HEP-1 cells after infection with LV-control or LV-FOXO1. The error bar represents the mean±SD of triplicate assays.(*p<0.05, **p<0.01; p-values were calculated using Student's t-test). **E.** Western blot analysis shows the expression of E-cadherin and N-cadherin in HCCLM3 and SK-HEP-1 cells that were transfected with siRNA-NC or siRNA-ZEB2. **F.** Downregulation of ZEB2 converted morphological changes of cells. **G.** The expression of FOXO1 and ZEB2 was detected in metastatic lung samples using immunohistochemistry. Scale bar indicates 200μm. **H.** Co-IP assays confirmed the co-localization of FOXO1 and ZEB2. Anti-FOXO1 or non-immune IgG was used to pull down FOXO1 from the total cell lysates. Anti-ZEB2 was used to detect ZEB2. **I.** ZEB2 promoter luciferase activity was inhibited by FOXO1. HCCLM3 cells were transfected with 40 ng of reporter construct, 500 ng of expression vector, and 5 ng of the internal control Renilla construct. The pGL3-basic plasmid was used as a negative control (left panel). Luciferase assays were performed 36h post transfection. pEGFP-NC group was set as 100% in each panel. The error bar represents the mean±SD of triplicate assays (***p<0.001). **J.** ChIP assay was used to analyze FOXO1 binding to the ZEB2 promoter in SK-HEP-1 cells. GAPDH and Twist1 are negative and positive control, respectively.

Snail, Slug, ZEB1, ZEB2, and Twist1 are key regulators of the EMT program. We examined the expression of these transcription factors to further investigate their potential interactions with FOXO1 during EMT. We found that endogenous mRNA levels of Snail1, Slug, ZEB1, ZEB2 and Twist1 were increased in FOXO1-silenced cells and were decreased in FOXO1-overexpressing cells (Figure 5A and 5C). A western blot analysis demonstrated similar results; however, Slug expression was not noticeably affected in the Huh7 cell line (Figure 5B and 5D). These findings suggest that FOXO1 might reverse EMT by interacting with EMT-associated transcription factors in HCC cells. According to our results, ZEB2 can promote EMT. We found that ZEB2 silencing enhanced the expression of E-cadherin and decreased the expression of N-cadherin (Figure 5E). In addition, ZEB2 silencing led to morphological changes in HCCLM3 and SK-HEP-1 cells (Figure 5F). We also found that FOXO1 and ZEB2 levels were inversely correlated and that ZEB2 was regulated by FOXO1 transcription. In addition, an immunohistochemistry analysis of metastatic nodules obtained from the nude mice revealed that FOXO1 overexpression inhibited ZEB2 expression (Figure 5G). Co-immunoprecipitation assays confirmed the interaction between FOXO1 and ZEB2 (Figure 5H). Based on the results that EMT transcriptional factors (including ZEB2) were decreased in FOXO1-overexpressing cells (Figure 5C and 5D), we hypothesized that FOXO1 might suppress ZEB2 promoter activity. ZEB2 promoter region was cloned into pGL3-Basic plasmid and the transcriptional regulation of FOXO1 was investigated through a dual-luciferase reporter assay. We first co-transfected pEGFP-FOXO1 or a negative control with pGL3-Basic control plasmid into HCCLM3 cells. As shown in Figure 5I (left panel), no difference on the pGL3-Basic control plasmid was found between pEGFP-FOXO1 and the negative control. However, pGL3-ZEB2 promoter activity was significantly inhibited by pEGFP-FOXO1 as shown in Figure 5I (right panel). These results demonstrated that ZEB2 promoter was inhibited by FOXO1. We subsequently investigated whether FOXO1 regulates ZEB2 expression directly by binding to specific sites within the ZEB2 promoter. ChIP assays confirmed that FOXO1 could directly bind to the ZEB2 promoter region in SK-HEP-1 cells (Figure 5J). Together, these data indicate that FOXO1 might reverse EMT in HCC, at least in part, via the direct inhibition of ZEB2.

## DISCUSSION

Characterizing the molecular mechanisms underlying HCC growth and metastasis is imperative to improve therapeutic outcomes. Down-regulation of FOXO1 has been detected in various cancers, and FOXO1 is down-regulated approximately 30-fold in HCC specimens compared with normal liver tissues. In addition, higher FOXO1 levels correlate with a more favorable prognosis [20, 21]. We demonstrated that FOXO1 plays a key role in repressing HCC invasion and metastasis. By overexpressing or silencing FOXO1 expression in HCC cells, we confirmed that FOXO1 can regulate the expression of EMT markers and EMT transcriptional activators. We also demonstrated that FOXO1 primarily inhibits EMT in HCC by down-regulating ZEB2. Furthermore, the development of tumor lung metastases was suppressed *in vivo* in nude mice injected with human HCC cells.

Metastasis is the leading cause of mortality in HCC. EMT is a vital step during the initiation of metastasis [4, 22], and TGF-β1 promotes cell migration, invasion, and metastasis by inducing EMT [23]. In this study, we found that TGF-β1 suppressed FOXO1 expression in HCC cells and that FOXO1 overexpression decreased the activity of the TGF-β-Smad signaling pathway. FOXO1 can inhibit the process of TGF-β-induced EMT. Thus, we speculated that a FOXO1-TGFβ feedback loop might represent a novel signaling pathway that regulates EMT and HCC progression. Multiple signaling molecules have been implicated in TGF-β-mediated EMT, including Smads, Erk, PI3K/Akt, RhoA and cofilin [24]. TGF-β can rapidly induce PI3K activation and Akt phosphorylation and can promote EMT, cell migration and survival. Previous studies have shown that phosphorylation of FoxO family members by activated Akt contributes to robust cell growth, proliferation and survival [25, 26]. In addition, some recent studies demonstrated that FOXO1 inhibits Akt activation, which may potentially inactivate TGF-β signaling [27, 28]. In summary, we hypothesized that the PI3K/Akt signaling pathway provides a link between TGF-β and FOXO1 in EMT in HCC cells.

Despite evidence that FOXO1 enhances cell invasion and migration, much less is known regarding the regulation of FOXO1 [29]. To our knowledge, this study is the first to report that FOXO1 plays a critical role in reversing EMT in HCC. Our findings suggest that the transcription factors Snail, Slug, ZEB1, ZEB2 and Twist1 play a role in FOXO1-inhibited metastasis and provide novel insights into the regulation of EMT. The results of this study are consistent with previous reports demonstrating that FOXO1 inhibits Twist1 mRNA expression in prostate cancer cells [30]. We focused our research on ZEB2 because ZEB2 expression was consistently affected by FOXO1 in all 4 HCC cell lines evaluated. Moreover, ZEB2 is highly expressed in lung metastatic nodules from HCC cells, and the overexpression of ZEB2 is associated with disease recurrence in HCC [31, 32]. As ZEB2 directly binds to the E-cadherin promoter and strongly inhibits E-cadherin expression, ZEB2 promotes tumor cell invasion and metastasis [33, 34]. In the present work, by immunohistochemistry and co-immunoprecipitation assays, we demonstrated an interaction between FOXO1 and ZEB2. A dual-luciferase reporter assay system and chromatin immunoprecipitation further confirmed that FOXO1 suppresses tumor cell invasion and metastasis mainly through direct binding to the ZEB2 promoter. This is the first report of a relationship between FOXO1 and ZEB2.

In summary, we identified a novel modulator of EMT in HCC, which acts in part by inhibiting EMT inducers. Thus, enhancing FOXO1 and repressing EMT inducers such as ZEB2 have potential clinical applications for HCC treatment approaches.

## MATERIALS AND METHODS

### Cells, lentiviral vectors and antibodies

The human HCC cell lines SMMC7721, Huh7, HCCLM3 and SK-HEP-1 were purchased from the American Type Culture Collection (ATCC) and were cultured in Dulbecco's Modified Eagle's Medium supplemented with 10% fetal bovine serum.

Cells were transfected with the following siRNA constructs (Genepharma, Shanghai, China): siScramble (random control sequence), sense: (5′-UU CUCCGAACGUGUCACGUTT-3′); or human FOXO1 siRNA, sense: (5′-GGAGGUAUGAGUC AGUAUATT-3′). Human ZEB2 siRNA, sence: (GGCAAGGCCUUCAAAUAUATT). Lentiviral vectors encoding the human FOXO1 gene were generated using the GV358 construct (Genechem, Shanghai, China), and the resulting construct is referred to as LV-FOXO1. An empty vector was used as the negative control, and this construct is referred to as the LV-control.

The antibodies against E-cadherin, β-catenin, Vimentin, N-cadherin, ZO-1, ZEB1, Snail, Slug and FOXO1 used for western blotting were obtained from Cell Signaling Technology (Beverly, MA, USA). The antibody against FOXO1 used for chromatin immunoprecipitation was obtained from Abcam (Cambridge, UK). The antibody against ZEB2 used for western blotting was obtained from Santa Cruz Biotechnology (Santa Cruz, CA, USA), and the antibody against ZEB2 used for the co-immunoprecipitation and immunohistochemistry assays was obtained from Abcam (Cambridge, UK).

### Transwell assays

Invasion and migration assays were conducted using Transwell chambers (BD Biosciences, SanJose, CA, USA). Cells were suspended in serum-free medium and seeded into the upper compartment of the chamber, and the lower compartment was filled with complete medium. The chamber was incubated for 24-72 h at 37°C with 5% carbon dioxide (CO_2_). For the invasion assay, polycarbonate membranes (8-μm pore size) in the upper compartment of 24-well Transwell culture chambers were coated with 5 mg/ml Matrigel (BD Biosciences). At the end of the incubation, the cells on the upper surface of the filter were removed by scrubbing. The invasive and migratory cells were stained with 0.5% crystal violet for 5 minutes and counted under a light microscope. Each assay was replicated 3 times.

### Wound-healing assay

Cell mobility was evaluated using a wound-healing assay. HCC cells were grown to full confluence in 6-well plates, and a wound was created by scratching the length of the well with a 10-μl pipette tip. Cells were treated with Mitomycin (20μg/ml) for 20 min. The cells were then washed 3 times with PBS and subsequently incubated with complete medium. Images were captured using an inverted digital camera at 0 h, 24 h and 48 h after the wound was generated. Using Image J software, cell migration was quantified by measuring the number of cells that migrated into the wound area at each time point. Each assay was replicated 3 times.

### Western blotting

The cells were harvested and lysed in RIPA buffer. Equal amounts of protein were separated on a sodium dodecyl sulfate-polyacrylamide gel electrophoresis (SDS-PAGE) gel and then transferred to a PVDF membrane. The membrane was incubated in blocking solution (5% milk/TBST) and subsequently incubated with the primary antibody overnight. The membrane was washed 3 times for 10 min with TBST and subsequently incubated with the HRP-conjugated secondary antibody for 1 or 2 h. Then, the membrane was washed 3 times for 10 min with TBST. Changes in protein expression were determined using the Chemiluminescent Substrate (Thermo Scientific).

### Quantitative real-time polymerase chain reaction (qRT-PCR)

Total cellular RNA was isolated using TRIzol (Invitrogen, Carlsbad, CA, USA) according to the manufacturer's recommendations. Total RNA (1 μg) was transcribed into cDNA using Reverse Transcriptase (Takara). The primer sequences are as follows:

SNAIL-F: TTCAACTGCAAATACTGCAACAAG

SNAIL-R: CAGTGTGGGTCCGGACATG

SNAIL2-F: TGGGCTGGCCAAACATAAG

SNAIL2-R: CCGCAGATCTTGCAAACACA

ZEB1-F: TCCATGCTTAAGAGCGGTAGCT

ZEB1-R: GTATCTTGTCTTTCATCCTGATTTCCA

ZEB2-F: TTCCTGGGCTACGACCATACC

ZEB2-R: CAAGCAATTCTCCCTGAAATCC

TWIST1-F: TGAGCAAGATTCAGACCCTCAA

TWIST1-R: CCATCCTCCAGACCGAGAAG

ACTB-F: GGGAAATCGTGCGTGACATT

ACTB-R: GGAACCGCTCATTGCCAAT

TGFB1-F: CGCCAGAGTGGTTATCTTTTGA

TGFB1-R:CGGTAGTGAACCCGTTGATGT

qRT-PCR was conducted using the FastStart Universal SYBR Green Master (ROX) and an Applied Biosystems 7500 Fast Real-Time PCR System.

### Immunofluorescence (IF)

HCC cells were seeded into wells and fixed in 4% paraformaldehyde after they had completely adhered to the wells. The cells were then washed 3 times for 10 min with PBS and blocked using normal goat serum with 0.1% Triton-100 for 60 min. The cells were incubated with the primary antibodies at 4°C overnight. Then, the cells were incubated with goat anti-rabbit secondary antibodies at room temperature for 60 min and counterstained with DAPI nuclear staining for 10 min before images were captured.

### Co-immunoprecipitation

Cells were lysed in co-immunoprecipitation buffer supplemented with protease inhibitor cocktail (Sigma-Aldrich) for 30 min. The lysates were centrifuged for 15 min at 12,000rpm at 4°C. The resulting supernatant was incubated with 1μg of IgG and 20 μl of Protein A/G beads (Santa Cruz, CA, USA) for 30 min at 4°C and centrifuged for 5 min at 1000 g at 4°C. Proteins were immunoprecipitated using the FOXO1 antibody or a control IgG antibody at 4°C for more than 4 h. Protein complexes were collected by incubating the reactions with 20 μl of immobilized Protein A/G beads at 4°C overnight. The collected protein complexes were washed 5 times with co-immunoprecipitation buffer and eluted by boiling in protein sample buffer under reducing conditions. The proteins were then resolved using SDS-PAGE and analyzed by western blotting.

### *In vivo* metastasis assay

BALB/c nude mice were obtained from the laboratory animal center of the Chinese Academy of Sciences, Shanghai. The experimental protocol was reviewed and approved by the Committee on the Use of Live Animals in Teaching and Research of the Harbin Medical University, Harbin, China. To evaluate lung metastasis, 2×10^6^ HCC cells were injected into nude mice via the tail vein. At 6 weeks after the injection, the mice (n=8/group) were anesthetized and intraperitoneally injected with 10 μl/g of D-luciferin (Caliper, Hopkinton, MA, USA). Bioluminescence was detected using a Berthold NIGHTOWL LB983 imaging machine. The mice were subsequently sacrificed, and the lungs were resected and fixed informaldehyde.

### Immunohistochemistry

Briefly, tissue samples were fixed in formaldehyde, embedded in paraffin, and sectioned prior to the immunostaining assays using antibodies against FOXO1 and ZEB2. Immunohistochemical staining was detected using a streptavidin-peroxidase complex.

### Luciferase assay

HCCLM3 cells were plated in 24-well plates and were co-transfected with 40 ng of reporter construct, 500 ng of expression vector, and 5 ng of the internal control Renilla construct (Promega, Madison, WI, USA) using Lipofectamine 2000 (Invitrogen). After 36 h, the luciferase activity was measured using a dual luciferase assay system (Promega, Madison, WI, USA).

### ChIP assay

Chromatin immunoprecipitation was performed using a ChIP Assay Kit (Beyotime, shanghai, China) according to manufacturer's protocol. Briefly, cells were cross-linked with 1% formaldehyde for 10 min. The cells were incubated at 37°C for 10 min to allow for the cross-linking of endogenous proteins and DNA. Then, 1.1 ml glycine solution (10×) was added to the medium, and the cells were incubated for 5 min at room temperature. The cells were then washed with cold PBS, scraped and collected on ice. Next, the cells were harvested, lysed and sonicated. After centrifugation, the supernatant was collected, and an equal amount of sonicated DNA fragments was immunoprecipitated with antibodies against FOXO1 or normal IgG (Santa Cruz, Santa Cruz, CA, USA) at 4°C overnight. Antibody–protein–DNA complexes were isolated by immunoprecipitation with protein A+G agarose beads. Following extensive washing, bound DNA fragments were eluted and amplified by PCR. The following PCR primer sequences were used: 5′- GCTTTAGGCACACATTCAAAG-3′, 5′-CAAATAAACTTCTAGCCTCACAGC-3′.

### Statistical analyses

The data are presented as the mean±SD. Differences between 2 groups were evaluated using Student's t-test. A value of p<0.05 was considered significant.
